# Densities, Viscosities of Pure 1-(2-Hydroxyethyl) Pyrrolidine, 3-Amino-1-Propanol, Water, and Their Mixtures at 293.15 to 363.15 K and Atmospheric Pressure

**DOI:** 10.1021/acs.jced.2c00648

**Published:** 2023-02-23

**Authors:** Ardi Hartono, Hanna K. Knuutila

**Affiliations:** Department of Chemical Engineering, University of Science and Technology, N-7491 Trondheim, Norway

## Abstract

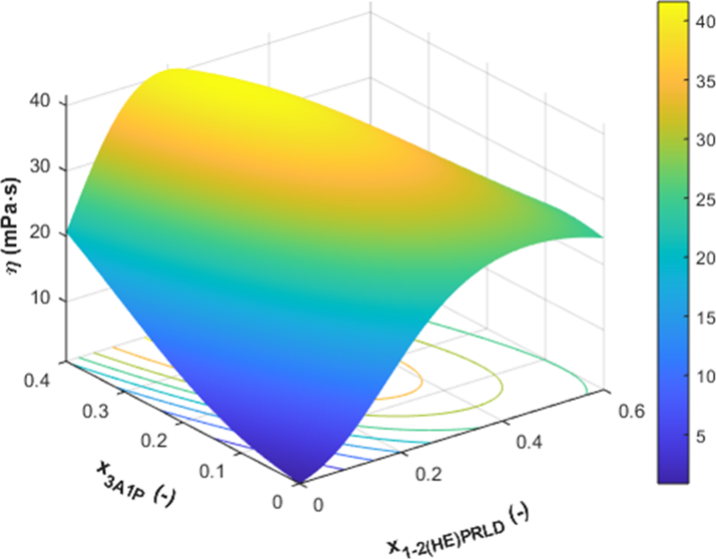

Densities and viscosities of pure 1-(2-hydroxyethyl) pyrrolidine, 3-amino-1-propanol, water, and their blends’ data are reported from 293.15 to 363.15 K and at ambient pressure. Densities of pure water show higher values than that of 3-amino-1-propanol and 1-(2-hydroxyethyl) pyrrolidine, whereas pure 3A1P is more viscous than 1-(2-hydroxyethyl) pyrrolidine and water. The excess molar volumes and viscosity deviations from the data are correlated to the Redlich–Kister equation. The shape and value for the excess molar volumes and viscosity deviations could explain the intermolecular interaction between the molecules.

## 1 Introduction

Lowering carbon dioxide emissions into the atmosphere is an urgent task to deploy. The power sector and cement industry, followed by the refinery sector, are the three biggest CO_2_ emission contributors.^1^ The absorption process with a chemically reactive solvent is a promising, mature, and viable technology to reduce CO_2_ emissions. A temperature swing absorption–desorption process is often used. In the process, gaseous CO_2_ contacts and reacts with an amine solvent to produce carbonated solvent in an absorber column. The carbonated solvent is regenerated in a stripper column to reverse the absorption reactions and to produce a pure gaseous CO_2_ stream by adding heat. Steam provides heat to reverse the chemical reactions and maintain the pressure in the stripper, making this technology energy-intensive.^2^ Finding better solvents is crucial. Solvent candidates should have high CO_2_ absorption rates, cyclic capacity, and equilibrium temperature sensitivity. They should be stable under operation with only a few side reactions forming degradation compounds and environmentally friendly.

1-(2-Hydroxyethyl) pyrrolidine is a strong bicarbonate-forming solvent for CO_2_ capture.^3−5^ However, 1-(2-hydroxyethyl) pyrrolidine, a tertiary amine, absorbs CO_2_ slowly. Therefore, a promoter (primary amines) is needed to increase the absorption rate.^6^ Basic physical properties (like density and viscosity) are essential to comprehensively understand the solvent performances. This work presents experimental density and viscosity data for 3-amino-1-propanol-promoted 1-(2-hydroxyethyl) pyrrolidine as a function of concentration and temperature. The measured data were used to calculate excess molar volumes and viscosity deviations. These two properties were then correlated with a Redlich–Kister (RK) model,^7^ representing the trends over different temperatures and concentrations. The generated correlations can be used when modeling absorption kinetics and vapor–liquid equilibrium.

Table 1 shows an overview of the literature on the density and viscosity of the two amines. For 3-amino-1-propanol data, experimental data for pure and aqueous solutions are available, but for aqueous 1-(2-hydroxyethyl) pyrrolidine solution, only limited data was found.^4^ Neither pure data were reported nor blends of these amines.

**Table 1:** Literature Data on Densities and Viscosities of 1-(2-Hydroxyethyl) Pyrrolidine and 3-Amino-1-propanol and Their Blends

no	data type	experiment conditions	reported data points	reference	remarks
3-Amino-1-propanol					
1	density	*T* = 293 to 333 K	5	(8)	Anton Paar DMA 4500
	viscosity	*x*_1_ > 0.99 (pure)	5		Ubbelohde viscometer
2	density	*T* = 293.15 K	1	(9)	Anton Paar DMA 60/602
		*x*_1_ > 0.99 (pure)			
3	density	*T* = 303.15 K	1	(10)	Densimeter DA-500E
		*x*_1_ > 0.99 (pure)			
4	density	*T* = 293 to 323 K	7	(11)	Anton Paar DSA 5000
		*x*_1_ > 0.99 (pure)			
5	density	*T* = 298 to 373 K	130	(12)	Anton Paar DMA 4500
		*x*_1_ = 0.027 to pure			
6	density	*T* = 293, 298, 303, 308 K	24	(13)	Anton Paar DSA 5000 M
		*x*_1_ = 0 to 0.016			
7	viscosity	*T* = 298 to 373 K	130	(14)	Anton-Paar Physica MCR 101 rheometer
		*x*_1_ = 0.027 to pure			
8	viscosity	*T* = 298.15 K	1	(15)	Anton Paar DSA 5000
		*x*_1_ = pure	1		Schott-Geräte AVS 350 Ubbelohde viscometer
9	density	*T* = 293 to 333 K	5	(16)	Anton Paar DMA 4500
	viscosity	*x*_1_ > 0.99 (pure)	5		Ubbelohde viscometer
1-(2-Hydroxyethyl) Pyrrolidine					
10	density	*T* = 298 to 333 K	7	(4)	Anton Paar DMA 4500
	viscosity	*x*_1_ > 0.11			Anton-Paar Physica MCR 101 rheometer

## 2 Experimental Section

### 2.1 Material and Methods

The chemicals listed in Table 2 were used directly without any further purification. Seven different concentrations of aqueous 1-(2-hydroxyethyl) pyrrolidine solutions, nine of aqueous 3-amino-1-propanol solutions, and five mixtures of 1-(2-hydroxyethyl) pyrrolidine and 3-amino-1-propanol were made. Approximately 50 g of the solutions was prepared gravimetrically using an analytical balance Mettler Toledo ME204. The capacity of the balance was from 0.16 mg to 220 g. The estimated combined expanded uncertainties were (i) for the scale  and (ii) for the prepared solutions  in mass fraction and  in mol fraction.

**Table 2:**
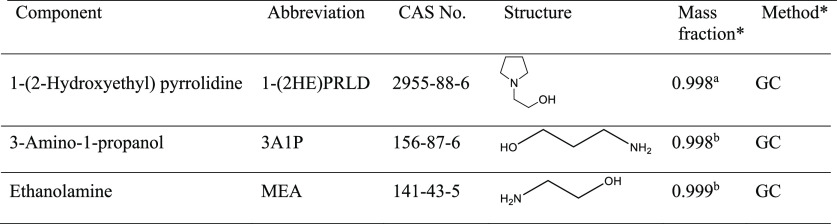
Chemical Used in This Work

a TCI Europe.

b Sigma-Aldrich. *Certificate of Analysis (CoA).

A DMA 4500 density meter coupled with a Lovis 2000ME viscosity meter, shown in Figure S1 (see in the Supporting Information), was used to measure the density and viscosity simultaneously. The DMA 4500 was calibrated by air and ultra-pure H_2_O supplied by the vendor at 298.15 K. The calibration was valid when the difference between the measured and reference was less than 0.03 kg·m^–3^. As the calibration at higher temperatures agreed well with the literature, no other temperature calibrations were done. The pressure was measured using DPI520 (Druck, Germany). The detailed procedure for the density measurement can be found in our previous work.^17^

The Lovis 2000ME micro-viscometer is based on a rolling ball principle, and operation conditions can be set from 278.15 to 373.15 K. A capillary borosilicate glass (with an internal diameter = 1.59 mm) is filled with a gold-coated stainless-steel ball to cover the viscosity measurements from 0.2 to 65 mPa·s. Ultra-pure H_2_O was used for calibration and was considered successful when the difference between the measured and reference was within 0.05% for at least five measurements.

The calibration results for water densities show good temperature and density repeatability (±0.01 K and ±0.03 kg·m^–3^) with the estimated expanded uncertainty *U*(ρ) of the measured density of about 0.5 kg·m^–3^. For water viscosities also, good repeatability (±0.01 K and ±0.05 mPa·s) was shown, with the estimated expanded uncertainty *U*(η) of about 0.08 mPa·s. In addition, two experiments with pure 3A1P at 298.15 K were performed using an Anton Paar MCR 100 rheometer^17^ to confirm the measured viscosity by the Lovis 2000ME micro viscometer.

### 2.2 Data Representation Using an RK Correlation

Excess molar volumes (*V*_*i*_^E^) can be expressed as
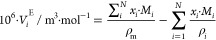
1where *x*_*i*_, *M*_*i*_, ρ_*i*_ and ρ_m_ are the mol fraction, molecular mass , and densities of pure substances and mixtures .

Viscosity deviations  as the difference between the pure substances and the (measured) viscosity of the blend are expressed as
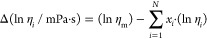
2where *x*_*i*_ is the mol fraction and η_*i*_ and η_m_ are the viscosities of pure substances and mixtures (mPa·s), respectively.

The required densities and viscosities of pure components in eqs 1 and 2 were also measured in this work. The measured data and literature data were regressed using eq 3 for density and eq 4 for viscosity, respectively
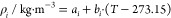
3
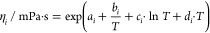
4

The excess molar volumes (*V*_*i*_^E^) and the viscosity deviations  as a function of temperature and concentration can be then correlated by the RK correlation^7^ and presented by eq 5 for the binary (*Y*_1,2_) and eq 6 for the ternary (*Y*_1,2,3_) systems as
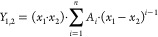
5
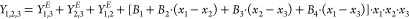
6

Temperature dependency of the RK equation is expressed as
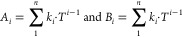
7where *Y* represents the excess molar volume or viscosity deviation for the considered binary or ternary system, *k*_*i*_ are the fitted parameters, *n* is the degree of correlation, and *T* is the temperature in Kelvin.

The parameters  in eqs 6 and 7 are fitted to the data by using an in-house Matlab code, a nonlinear fitting model where a root means square error (RMSE)^18^ was minimized as
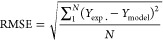
8

The quality of the fit was also expressed by an average absolute relative (AARD) deviation as
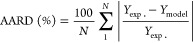
9where *N* is the number of the density and viscosity data. *Y*_exp._ is the excess molar volume (eq 1) and the viscosity deviations (eq 2), and *Y*_model_ are the RK model results (eqs 5 to 7).

## 3 Results and Discussion

### 3.1 Setup Calibration and Verification

The water calibration results are presented in the Supporting Information (Figure S2). The agreement with the literature was good, with the AARD values of 0.02% for density and 1.2% for viscosity. To further verify the setup, densities and viscosities of 30 mass % of MEA  were studied (see Table S1 in the Supporting Information) and compared to the literature^17^ (see Figure S3 for data comparison and Figure S4 for deviations). For viscosity, the data in this work are mainly from a Lovis 2000ME micro viscometer while the literature data are based on Anton Paar MCR 100 rheometer. Still, the agreement was good for 30 mass % of MEA, with AARD of 0.2% for viscosity. For density, the AARD was 0.03%.

### 3.2 Density and Viscosity Results

The measured densities and viscosities of three binary and one ternary system at different concentrations and temperatures are reported in Tables 3–6 for 1-(2HE)PRLD(1)/H_2_O(3), 3A1P(2)/H_2_O(3), 1-(2HE)PRLD(1)/3A1P(2) and a ternary of 1-(2HE)PRLD(1)/3A1P(2)/H_2_O(3), respectively. All measurements were performed twice, and the average values are given in all tables. The repeatability was good (within  for density and ±0.22 mPa·s for viscosity).

**Table 3:** Densities, Excess Molar Volumes, Viscosities, and Viscosity Deviations of Binary 1-(2HE)PRLD(1)/H_2_O(3) as a Function of mol Fraction (*x*_i_) and Temperature at Ambient Pressure (101.0 kPa)^a^

*x*_1_	ρ_13_ (kg·m^–3^)	10^6^·*V*_13_^E^ (m^3^·mol^–1^)	η_13_ (mPa·s)	(mPa·s)
293.15 K				
0.0000	998.20		0.99	
0.0490	1010.67	–0.40	3.32	1.08
0.0940	1015.83	–0.69	7.03	1.71
0.1892	1015.46	–1.07	18.06	2.41
0.3827	1005.42	–1.30	37.36	2.63
0.5858	994.12	–1.08	33.51	2.00
0.7492	986.60	–0.70	23.58	1.22
0.8317	983.39	–0.47	19.37	0.81
1.0000	978.18		13.31	
298.15 K				
0.0000	997.05		0.89	
0.0490	1008.29	–0.39	2.75	1.01
0.0940	1012.54	–0.67	5.58	1.61
0.1892	1011.62	–1.04	13.67	2.26
0.3827	1001.41	–1.27	27.40	2.47
0.5858	990.32	–1.07	25.13	1.88
0.7492	982.68	–0.69	18.31	1.15
0.8317	979.46	–0.46	15.29	0.77
1.0000	974.30		10.83	
303.15 K				
0.0000	995.69		0.80	
0.0490	1005.89	–0.38	2.34	0.96
0.0940	1009.40	–0.65	4.62	1.53
0.1892	1007.83	–1.01	10.82	2.15
0.3827	997.53	–1.26	20.75	2.33
0.5858	985.88	–1.02	19.12	1.76
0.7492	978.96	–0.70	14.52	1.09
0.8317	975.87	–0.49	12.41	0.73
1.0000	970.39		8.96	
313.15 K				
0.0000	992.29		0.66	
0.0490	1000.31	–0.36	1.73	0.86
0.0940	1002.35	–0.61	3.18	1.37
0.1892	999.86	–0.96	6.85	1.92
0.3827	989.05	–1.20	12.40	2.08
0.5858	977.99	–1.01	11.85	1.57
0.7492	970.89	–0.69	9.45	0.97
0.8317	967.67	–0.47	8.23	0.65
1.0000	962.37		6.29	
323.15 K				
0.0000	988.15		0.55	
0.0490	994.25	–0.35	1.34	0.79
0.0940	995.05	–0.58	2.35	1.25
0.1892	991.56	–0.91	4.69	1.74
0.3827	980.52	–1.15	7.90	1.85
0.5858	969.59	–0.98	7.69	1.39
0.7492	962.63	–0.67	6.43	0.87
0.8317	959.45	–0.45	5.75	0.58
1.0000	954.23		4.60	
333.15 K				
0.0000	983.35		0.47	
0.0490	987.72	–0.33	1.07	0.72
0.0940	987.48	–0.55	1.79	1.15
0.1892	983.01	–0.86	3.37	1.59
0.3827	971.91	–1.11	5.34	1.66
0.5858	961.07	–0.94	5.29	1.25
0.7492	954.13	–0.63	4.55	0.77
0.8317	951.16	–0.44	4.18	0.52
1.0000	946.03		3.46	
343.15 K				
0.0000	977.99		0.41	
0.0490	980.76	–0.31	0.89	0.68
0.0940	979.61	–0.52	1.41	1.06
0.1892	974.28	–0.82	2.52	1.46
0.3827	963.04	–1.06	3.76	1.50
0.5858	952.44	–0.91	3.79	1.12
0.7492	945.58	–0.61	3.36	0.69
0.8317	942.69	–0.43	3.12	0.46
1.0000	937.65		2.69	
353.15 K				
0.0000	972.04		0.36	
0.0490	973.40	–0.30	0.74	0.62
0.0940	971.47	–0.50	1.15	0.98
0.1892	965.54	–0.78	1.93	1.34
0.3827	953.83	–1.01	2.79	1.36
0.5858	943.49	–0.87	2.82	1.01
0.7492	936.87	–0.59	2.58	0.64
0.8317	934.04	–0.41	2.45	0.44
1.0000	929.16		2.13	
363.15 K				
0.0000	965.85		0.34	
0.0490	965.75	–0.29	0.64	0.55
0.0940	963.03	–0.47	0.96	0.89
0.1892	956.40	–0.74	1.53	1.20
0.3827	944.45	–0.95	2.14	1.21
0.5858	934.40	–0.83	2.18	0.89
0.7492	928.09	–0.57	2.06	0.57
0.8317	925.32	–0.39	1.94	0.38
1.0000	920.60		1.76	

a Standard uncertainties *u* are *u* (*P*) = 0.3 kPa, *u* (*T*) = 0.01 K, , *u* (η) = 0.3 mPa·s for η ≤ 10 mPa·s and *u* (η) = 0.6 mPa·s for η > 10 mPa·s, , *U*_C_ (Δ(η)) = 0.05 mPa·s, and *U*_C_ (ρ) = 0.8 kg·m^–3^, with a 0.95 level of confidence (*k* ≈ 2).

**Table 4:** Densities, Excess Molar Volumes, Viscosities, and Viscosity Deviations of Binary 3A1P(2)/H_2_O(3) as a Function of mol Fraction (*x*_i_) and Temperature at Ambient Pressure (101.0 kPa)^a^

*x*_2_	ρ_23_ (kg/m^3^)	10^6^·*V*_23_^E^ (m^3^/mol)	η_23_ (mPa·s)	(mPa·s)
293.15 K				
0.0000	998.20		0.99	
0.0263	999.42	–0.05	1.45	0.29
0.0565	1002.04	–0.13	2.19	0.59
0.0926	1005.78	–0.25	3.44	0.91
0.1368	1010.00	–0.41	5.66	1.25
0.1916	1013.64	–0.60	9.48	1.57
0.2643	1015.42	–0.78	15.97	1.83
0.3563	1014.06	–0.89	24.62	1.94
0.4891	1008.88	–0.89	34.10	1.79
0.6804	1000.11	–0.66	39.27	1.24
1.0000	987.52		35.54	
298.15 K				
0.0000	997.05		0.88	
0.0263	998.08	–0.05	1.27	0.28
0.0565	1000.32	–0.13	1.88	0.57
0.0926	1003.60	–0.25	2.88	0.87
0.1368	1007.27	–0.40	4.61	1.19
0.1916	1010.47	–0.58	7.53	1.49
0.2643	1011.83	–0.76	12.33	1.73
0.3563	1010.26	–0.87	18.66	1.83
0.4891	1004.98	–0.87	25.55	1.69
0.6804	996.20	–0.65	29.74	1.19
1.0000	983.61		27.10	
303.15 K				
0.0000	995.69		0.80	
0.0263	996.55	–0.05	1.13	0.26
0.0565	998.43	–0.13	1.64	0.53
0.0926	1001.28	–0.25	2.47	0.82
0.1368	1004.47	–0.40	3.85	1.12
0.1916	1007.22	–0.57	6.13	1.41
0.2643	1008.27	–0.74	9.83	1.64
0.3563	1006.52	–0.86	14.64	1.74
0.4891	1001.21	–0.86	20.01	1.61
0.6804	992.40	–0.65	23.07	1.13
1.0000	979.62		21.36	
313.15 K				
0.0000	992.25		0.67	
0.0263	992.78	–0.05	0.91	0.22
0.0565	994.06	–0.13	1.27	0.47
0.0926	996.16	–0.24	1.85	0.74
0.1368	998.54	–0.38	2.77	1.01
0.1916	1000.47	–0.55	4.20	1.26
0.2643	1000.89	–0.71	6.44	1.47
0.3563	998.79	–0.82	9.31	1.56
0.4891	993.34	–0.83	12.54	1.45
0.6804	984.66	–0.65	14.51	1.02
1.0000	971.69		13.68	
323.15 K				
0.0000	988.09		0.55	
0.0263	988.32	–0.06	0.75	0.24
0.0565	989.13	–0.13	1.02	0.46
0.0926	990.59	–0.24	1.44	0.70
0.1368	992.25	–0.37	2.08	0.94
0.1916	993.47	–0.53	3.02	1.16
0.2643	993.33	–0.69	4.45	1.35
0.3563	991.05	–0.80	6.31	1.44
0.4891	985.34	–0.81	8.24	1.33
0.6804	976.72	–0.64	9.55	0.94
1.0000	963.69		9.19	
333.15 K				
0.0000	983.29		0.48	
0.0263	983.25	–0.06	0.63	0.20
0.0565	983.69	–0.13	0.84	0.41
0.0926	984.59	–0.24	1.16	0.64
0.1368	985.62	–0.36	1.61	0.85
0.1916	986.23	–0.51	2.28	1.06
0.2643	985.65	–0.66	3.22	1.22
0.3563	983.15	–0.78	4.41	1.29
0.4891	977.27	–0.79	5.70	1.20
0.6804	968.54	–0.62	6.57	0.85
1.0000	955.74		6.48	
343.15 K				
0.0000	977.92		0.42	
0.0263	977.68	–0.06	0.54	0.18
0.0565	977.73	–0.13	0.71	0.38
0.0926	978.17	–0.23	0.95	0.59
0.1368	978.65	–0.35	1.29	0.79
0.1916	978.74	–0.49	1.77	0.97
0.2643	977.77	–0.64	2.43	1.11
0.3563	975.08	–0.75	3.24	1.18
0.4891	969.04	–0.76	4.11	1.09
0.6804	960.48	–0.60	4.74	0.77
1.0000	947.90		4.77	
353.15 K				
0.0000	972.04		0.36	
0.0263	971.62	–0.06	0.46	0.19
0.0565	971.28	–0.13	0.60	0.38
0.0926	971.35	–0.23	0.80	0.59
0.1368	971.39	–0.34	1.06	0.76
0.1916	971.04	–0.48	1.42	0.93
0.2643	969.72	–0.62	1.89	1.05
0.3563	966.63	–0.72	2.49	1.11
0.4891	960.70	–0.73	3.13	1.04
0.6804	952.29	–0.58	3.54	0.72
1.0000	939.95		3.60	
363.15 K				
0.0000	965.85		0.34	
0.0263	965.35	–0.06	0.42	0.14
0.0565	964.43	–0.13	0.53	0.32
0.0926	964.16	–0.22	0.69	0.50
0.1368	963.84	–0.33	0.89	0.67
0.1916	963.06	–0.46	1.16	0.82
0.2643	961.45	–0.59	1.51	0.93
0.3563	958.20	–0.69	1.93	0.97
0.4891	952.31	–0.71	2.39	0.90
0.6804	944.13	–0.58	2.74	0.63
1.0000	931.88		2.88	

a Standard uncertainties *u* are *u* (*P*) = 0.3 kPa, *u* (*T*) = 0.01 K, , *u* (η) = 0.3 mPa·s for η ≤ 10 mPa·s and *u* (η) = 0.6 mPa·s for η > 10 mPa·s, , *U*_C_ (Δ(η)) = 0.05 mPa·s, and *U*_C_ (ρ) = 0.8 kg·m^–3^, with a 0.95 level of confidence (*k* ≈ 2).

**Table 5:** Densities, Excess Molar Volumes, Viscosities, and Viscosity Deviations of Binary 1-(2HE)PRLD(1)/3A1P(2) as a Function of mol Fraction (*x*_i_) and Temperature at Ambient Pressure (101.0 kPa)^a^

*x*_1_	ρ_12_ (kg/m^3^)	10^6^·*V*_12_^E^ (m^3^/mol)	η_12_ (mPa·s)	(mPa·s)
293.15 K				
0.0000	987.52		35.48	
0.0679	986.75	–0.01	35.01	0.05
0.2181	985.19	–0.04	33.21	0.15
0.3941	983.40	–0.05	29.34	0.20
0.6031	981.59	–0.06	23.68	0.19
0.8549	979.49	–0.04	16.81	0.09
1.0000	978.19		13.31	
298.15 K				
0.0000	983.62		27.13	
0.0679	982.80	–0.01	26.88	0.05
0.2181	981.27	–0.04	25.38	0.13
0.3941	979.51	–0.05	22.54	0.18
0.6031	977.70	–0.06	18.44	0.17
0.8549	975.63	–0.04	13.45	0.09
1.0000	974.33		10.81	
303.15 K				
0.0000	979.73		21.45	
0.0679	979.06	–0.02	21.18	0.05
0.2181	977.46	–0.05	20.00	0.12
0.3941	975.61	–0.05	17.75	0.16
0.6031	973.86	–0.06	14.73	0.15
0.8549	971.77	–0.04	10.96	0.07
1.0000	970.50		8.96	
313.15 K				
0.0000	971.82		13.66	
0.0679	971.46	–0.05	13.59	0.05
0.2181	969.42	–0.04	12.70	0.10
0.3941	967.71	–0.06	11.45	0.13
0.6031	965.80	–0.06	9.65	0.12
0.8549	963.81	–0.05	7.50	0.06
1.0000	962.46		6.30	
323.15 K				
0.0000	964.06		9.28	
0.0679	964.39	–0.11	9.07	0.03
0.2181	961.43	–0.03	8.51	0.06
0.3941	959.70	–0.05	7.74	0.09
0.6031	957.78	–0.06	6.66	0.09
0.8549	955.74	–0.05	5.35	0.04
1.0000	954.35		4.65	
333.15 K				
0.0000	955.89		6.52	
0.0679	955.31	–0.04	6.43	0.03
0.2181	953.39	–0.04	6.01	0.06
0.3941	951.20	–0.02	5.45	0.07
0.6031	949.59	–0.06	4.79	0.07
0.8549	947.45	–0.04	3.95	0.04
1.0000	946.14		3.47	
343.15 K				
0.0000	948.06		4.83	
0.0679	947.49	–0.04	4.68	0.01
0.2181	945.51	–0.05	4.41	0.04
0.3941	943.07	–0.02	4.04	0.05
0.6031	941.37	–0.06	3.63	0.07
0.8549	939.19	–0.05	3.02	0.03
1.0000	937.76		2.69	
353.15 K				
0.0000	940.01		3.60	
0.0679	939.51	–0.05	3.55	0.02
0.2181	937.45	–0.06	3.35	0.04
0.3941	934.89	–0.02	3.06	0.04
0.6031	933.13	–0.06	2.79	0.06
0.8549	930.80	–0.04	2.36	0.03
1.0000	929.49		2.13	
363.15 K				
0.0000	931.96		2.81	
0.0679	931.69	–0.08	2.78	0.02
0.2181	929.43	–0.08	2.61	0.03
0.3941	926.72	–0.03	2.40	0.04
0.6031	924.82	–0.06	2.22	0.06
0.8549	922.38	–0.03	1.91	0.03
1.0000	921.04		1.73	

a Standard uncertainties *u* are *u* (*P*) = 0.3 kPa, *u* (*T*) = 0.01 K, , *u* (η) = 0.3 mPa·s for η ≤ 10 mPa·s and *u* (η) = 0.6 mPa·s for η > 10 mPa·s, , *U*_C_ (Δ(η)) = 0.05 mPa·s, and *U*_C_ (ρ) = 0.8 kg·m^–3^, with a 0.95 level of confidence (*k* ≈ 2).

**Table 6:** Densities, Excess Molar Volumes, Viscosities, and Viscosity Deviations of Ternary 1-(2HE)PRLD(1)/3A1P(2)/H_2_O(3) as a Function of mol Fraction (*x*_i_) and Temperature at Ambient Pressure (101.0 kPa)^a^

*x*_1_	*x*_2_	ρ_123_ (kg/m^3^)	10^6^·*V*_123_^E^ (m^3^/mol)	η_123_ (mPa·s)	(mPa·s)
293.15 K					
0.0189	0.0320	1005.71	–0.23	2.51	0.76
0.5063	0.0991	994.83	–1.03	35.99	1.92
0.0504	0.6185	999.74	–0.71	40.02	1.35
0.0831	0.3820	1008.43	–0.99	36.23	2.02
0.1148	0.0667	1016.89	–0.93	14.48	2.14
298.15 K					
0.0189	0.0320	1003.78	–0.23	2.15	0.73
0.5063	0.0991	990.88	–1.01	27.06	1.82
0.0504	0.6185	995.80	–0.70	30.33	1.29
0.0831	0.3820	1004.52	–0.98	27.35	1.92
0.1148	0.0667	1013.22	–0.91	11.13	2.02
303.15 K					
0.0189	0.0320	1001.69	–0.23	1.85	0.69
0.5063	0.0991	986.92	–1.00	20.49	1.70
0.0504	0.6185	991.91	–0.70	23.29	1.22
0.0831	0.3820	1000.60	–0.96	20.85	1.81
0.1148	0.0667	1009.49	–0.88	8.81	1.90
313.15 K					
0.0189	0.0320	996.94	–0.22	1.41	0.61
0.5063	0.0991	978.77	–0.97	12.64	1.51
0.0504	0.6185	983.92	–0.68	14.45	1.10
0.0831	0.3820	992.64	–0.93	12.87	1.62
0.1148	0.0667	1001.78	–0.84	5.76	1.70
323.15 K					
0.0189	0.0320	991.64	–0.22	1.11	0.58
0.5063	0.0991	971.25	–1.01	8.15	1.34
0.0504	0.6185	975.85	–0.66	9.47	0.99
0.0831	0.3820	984.54	–0.90	8.38	1.47
0.1148	0.0667	993.88	–0.80	3.99	1.55
333.15 K					
0.0189	0.0320	985.84	–0.21	0.91	0.51
0.5063	0.0991	962.03	–0.92	5.60	1.20
0.0504	0.6185	967.75	–0.64	6.56	0.90
0.0831	0.3820	976.35	–0.88	5.76	1.32
0.1148	0.0667	985.80	–0.76	2.90	1.40
343.15 K					
0.0189	0.0320	979.53	–0.21	0.76	0.48
0.5063	0.0991	953.41	–0.89	4.00	1.08
0.0504	0.6185	959.56	–0.62	4.69	0.81
0.0831	0.3820	968.00	–0.85	4.15	1.21
0.1148	0.0667	977.51	–0.73	2.22	1.29
353.15 K					
0.0189	0.0320	972.81	–0.21	0.65	0.48
0.5063	0.0991	944.65	–0.86	2.96	0.98
0.0504	0.6185	951.28	–0.60	3.50	0.76
0.0831	0.3820	959.55	–0.82	3.11	1.13
0.1148	0.0667	969.02	–0.70	1.72	1.21
363.15 K					
0.0189	0.0320	965.67	–0.20	0.56	0.40
0.5063	0.0991	935.82	–0.83	2.30	0.88
0.0504	0.6185	943.15	–0.60	2.73	0.70
0.0831	0.3820	951.01	–0.80	2.40	1.01
0.1148	0.0667	960.30	–0.67	1.39	1.08

a Standard uncertainties *u* are *u* (*P*) = 0.3 kPa, *u* (*T*) = 0.01 K, , *u* (η) = 0.3 mPa·s for η ≤ 10 mPa·s and *u* (η) = 0.6 mPa·s for η > 10 mPa·s, , *U*_C_ (Δ(η)) = 0.05 mPa·s, and *U*_C_ (ρ) = 0.8 kg·m^–3^, with 0.95 level of confidence (*k* ≈ 2).

### 3.3 Regressed Correlations of the Pure Amines, Water, and Its Blends

**Figure 1:**
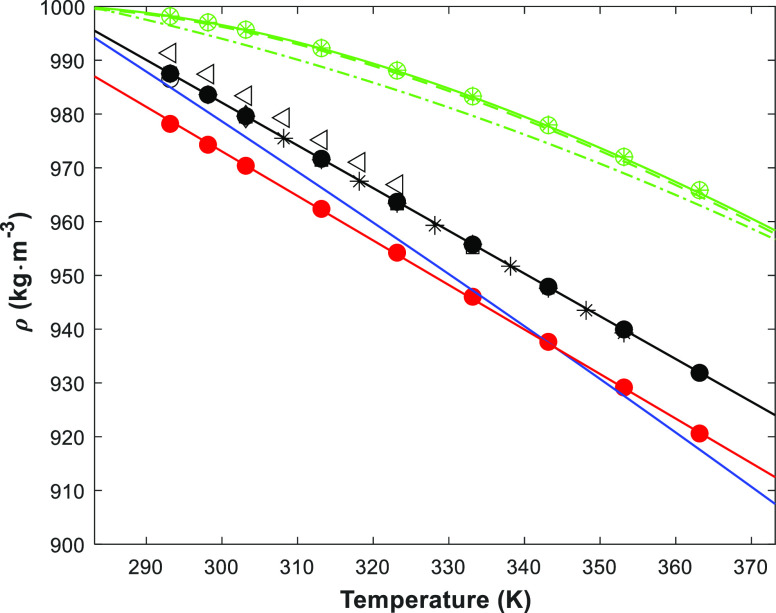
Density of pure components (points, exp.; lines, correlations) (1-(2HE)PRLD: red ●/red —, this work); (3A1P: ●/—, this work; ◯,^9^ □,^16^ ◇,^10^ black 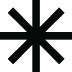,^12^ ◁,^11^ blue —,^19^) (H_2_O: green ◯/green 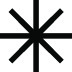, this work; green •-•,^19^ green ---,^20^ green —,^21^).

Figure 1 shows the density of the pure amines and water, and the density correlation (eq 3). The fitted parameters are given in Table S2 in the Supporting Information As shown in Table 1, no density data for pure 1-(2HE)PRLD was found, but some data for pure 3A1P were available. Figure 1 shows good agreement of this work with the literature, except for one data set^11^ where the reported data seem to be consistently higher. The chemical purity may explain the difference. Different correlations for the pure components are also presented. The literature data for 3A1P (solid blue line) and H_2_O (green dashed-dot line)^19^ underpredict the data, but the proposed correlation by Jones and Harris^20^ for H_2_O fits well with the measured data in this work.

**Figure 2:**
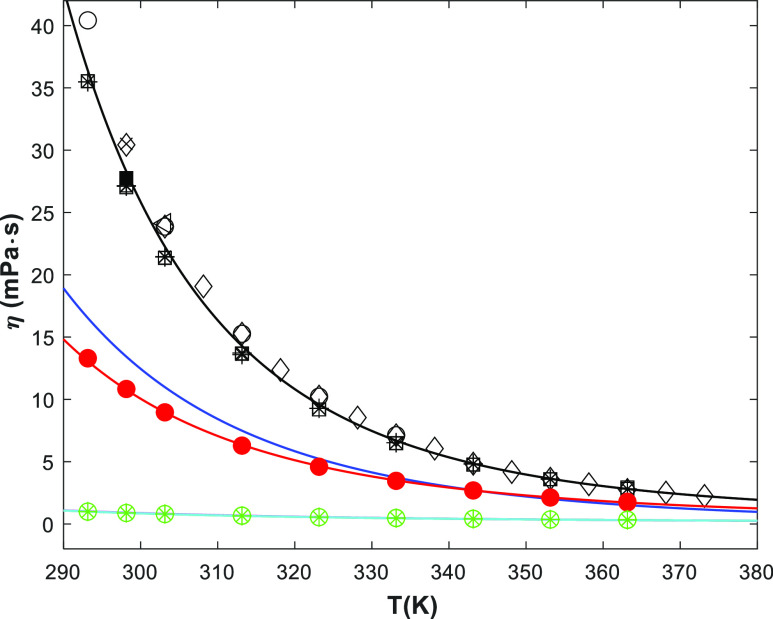
Viscosity of pure components (points, exp; lines, correlation) (1-(2HE)PRLD: red ●/red —, this work) (3A1P: 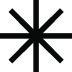/□/■/—, this work; ◯,^16^ ◁,^10^ ◇,^14^ ×,^15^ blue —,^19^) (H_2_O: green ◯/green 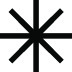, this work; green —,^22^ pink —,^19^ sky blue —,^12,23^).

Similarly, Figure 2 shows the viscosity of the pure amines and water, and the viscosity correlation (eq 4). The fitted parameters are given in Table S2 (in the Supporting Information). No literature data were found for pure 1-(2HE)PRLD, but some data for pure 3A1P were available. Figure 2 shows that the 3A1P results from this work agree well at higher temperatures (*T* ≥ 323 K) with literature data^10,14−16^ but tend to be lower at lower temperatures (*T* < 323 K). The lowest temperature has the highest deviation. To verify our data, two experiments at 298.15 K were performed using a different apparatus (Anton Paar MCR 100 rheometer), and the results (■) fit better to this work (□/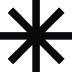) than other literature data. Thus, it can be speculated if the chemical purity may explain the differences between this work and the literature. The viscosity correlations available in the literature for 3A1P (solid blue line) and H_2_O (magenta line)^19^ are also presented. However, the 3A1P data are underpredicted by the literature while the agreement is better for water. The water correlations agree well with the proposed correlation by Kestin et al.^22^ and with the results in this work.

**Figure 3:**
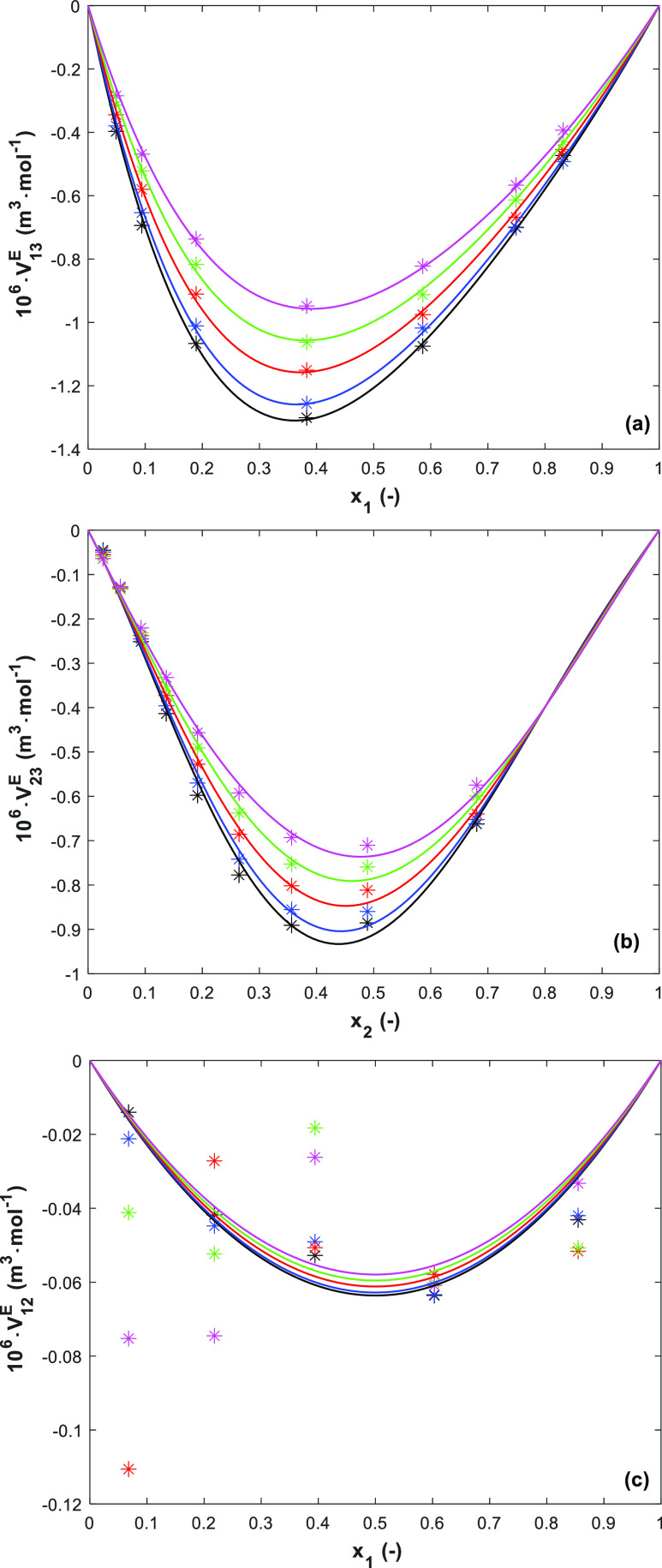
Excess molar volumes for (a) binary 1-(2HE)PRLD(1)/H_2_O(3), (b) binary 3A1P(2)/H_2_O(3), and (c) binary 1-(2HE)PRLD(1)/3A1P(2) at *T* = 293.15 to 363.15 K and at ambient pressure (points; calculated from the measured data (eq 1); lines, the RK prediction (eq 5); —, 293.15 K; blue —, 303.15 K; red —, 323.15 K; green —, 343.15 K; pink —, 363.15).

The *V*^E^ values in Tables 3–6 were regressed using the RK model (eq 5), and the fitted parameters are given in Table 7, along with the statistical information related to the fit (RMSE, AARD, uncertainties of the fitted parameter () and *p*-values). The *p*-value described the results from the statistical hypothesis test and can indicate whether the required correlations (eqs 5 and 6) are overparameterized (i.e., if the *p*-value ≥ 0.1). As given in Table 7, different numbers of parameters are required for each system: for the 1-(2HE)PRLD (1)/H_2_O(3) system, six parameters are required, whereas eight parameters are needed for the 3A1P(2)/H_2_O(3) system. The presentation of the *V*^E^ data and the RK correlation are shown, respectively, in Figure 3a for 1-(2HE)PRLD(1)/H_2_O(3), 5(b) for 3A1P(2)/H_2_O(3), and 5(c) for 1-(2HE)PRLD(1)/3A1P(2).

**Table 7:** Fitted RK Parameters for the Excess Molar Volumes

coefficient		1-(2HE)PRLD(1)/H_2_O(3)		3A1P(2)/H_2_O(3)		1-(2HE)PRLD(1)/3A1P(2)		1-(2HE)PRLD(1)/3A1P(2)/H_2_O(3)	
*A*_*i*_/*B*_*i*_	*k*_*i*_	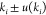	*p*_value_^*i*^	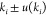	*p*_value_^*i*^	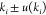	*p*_value_^*i*^	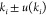	*p*_value_^*i*^
1	1	–9.74 ± 0.1	0.0000	–6.62 ± 0.2	0.0000	–0.35 ± 0.2	0.0354	–3.45 ± 0.7	0.0000
	2	0.01677 ± 0.0004	0.0000	0.01013 ± 0.0007	0.0000	–0.00032 ± 0.0005	0.5000		
2	3	7.72 ± 0.2	0.0000	5.70 ± 0.8	0.0000				
	4	–0.0170 ± 0.001	0.0000	–0.0148 ± 0.002	0.0000				
3	5	–3.49 ± 0.5	0.0000	5.19 ± 0.8	0.0000				
	6	0.0078 ± 0.001	0.0000	–0.0124 ± 0.003	0.0000				
4	7			–5.4 ± 2	0.0023				
	8			0.0150 ± 0.005	0.0062				
RMSE		0.04		0.03		0.02		0.04	
AARD (%)		0.04		0.04		0.03		0.098	

From a liquid theory,^24^ the sign of the *V*^E^ values can be negative or positive compared to their pure liquid component and can be attributed from physical, chemical, and structural characteristics. Physical contribution (like dispersion forces and non-specific physical interactions) is usually weak and gives a positive value. Chemical contribution and structural characteristics can both give positive and negative values. The positive value in the chemical contribution is associated with the breaking up in pure liquid components. However, negative values for specific interactions, such as the formation of hydrogen bonding, charge transfer, and dipole–dipole interactions, are common.^25^ The positive and negative values for the structural characteristic are due to the favorable and unfavorable geometrical fitting of the molecular structure (e.g., size, shape, and volume).

The *V*^E^ of the studied systems show negative values (relative to the ideal ones obtained from the corresponding mol fraction and density of pure components in eq 1). The negative *V*^E^ indicates a volume contraction in the real mixture, which can be attributed to the chemical and structural characteristic contributions. As alkanolamine, 1-(2HE)PRLD and 3A1P have two different functional groups (see Table 1), i.e., hydroxyl (−OH) and amino (−N or −NH_2_) groups, in their molecular structure. It is the same case in water (−H and −OH). The (−OH) group can function as a hydrogen bond donor and acceptor, while the amino groups function as proton donors. As a tertiary amine, 1-(2HE)PRLD, the amino (−N) group usually gives a stronger base than the amino (−NH_2_) group of the primary amine in 3A1P.^3^ A stronger base creates stronger hydrogen bonding in the presence of H_2_O. This could be the reason why the mixture of 1-(2HE)PRLD(1)/H_2_O (3) shows more volume contraction than in the 3A1P(2)/H_2_O(3) system (Figure 3a,b). A comparison with literature data^12,13^ for 3A1P(2)/H_2_O(3) is shown in Figures S5 in the Supporting Information In aqueous solutions, molecular structures of 1-(2HE)PRLD and 3A1P can also contribute to the negative value as a consequence of their structural characteristic for favorable geometrical fitting. The *V*^E^ increases with temperature, and it can be interpreted as a sign that the interactional effects are dominant. For blends of the two amines, the *V*^E^ show low negative values with a clear trend at lower temperatures (*T* ≤ 303 K) but larger scatter at higher temperatures (*T* > 303 K). 1-(2HE)PRLD has cyclic pyrrolidine and hydroxyethyl (-C_2_H_5_O) groups^26^ and 3A1P has an aminopropanol group.^27^ It may be the consequence of the mixture being favorable for geometric filling due to steric hindrances at lower temperatures but then to be an unfavorable structural geometric at higher temperatures due to molecular motion (Figure 3c). The two binary aqueous solutions show the largest negative *V*^E^ values, indicating the most efficient packing^28^ in the system, but for blends of the two amines, the deviations were smaller.

**Figure 4:**
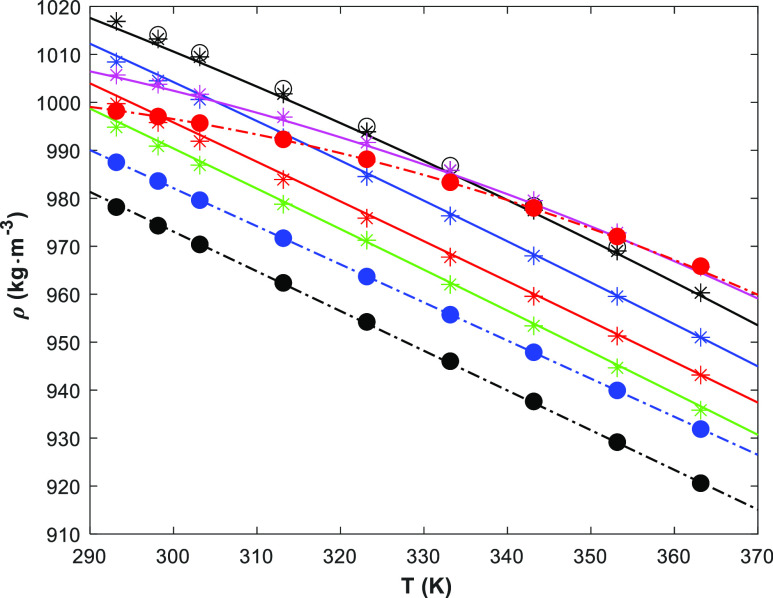
Densities of ternary 1-(2HE)PRLD(1)/3A1P(2)/H_2_O(3) at 293.15 to 363.15 K and at ambient pressure (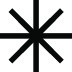/◯/—, ; blue 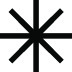/blue —, ; red 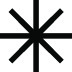/red —, ; green 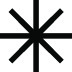/green —, ; pink 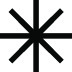/pink —, ; ●/•-•, *x*_1_ = 1.0000; blue ●/blue •-•, *x*_2_ = 1.0000; red ●/red •-•, *x*_3_ = 1.0000; lines, eq 6).

The representations of the RK model for the density data for the binary systems are presented in the Supporting Information (see Figures S6–S8). The reported densities data at low concentrations^13^ seem to fit better to this work than the reported data from the literature^12^ (see Figures S5 and S7). The results in the case of the ternary 1-(2HE)PRLD(1)/3A1P(2)/H_2_O(3) system are shown in Figure 4, while Figure S9 in the Supporting Information shows the parity plot and ratio between the calculated and the measured densities.

**Figure 5:**
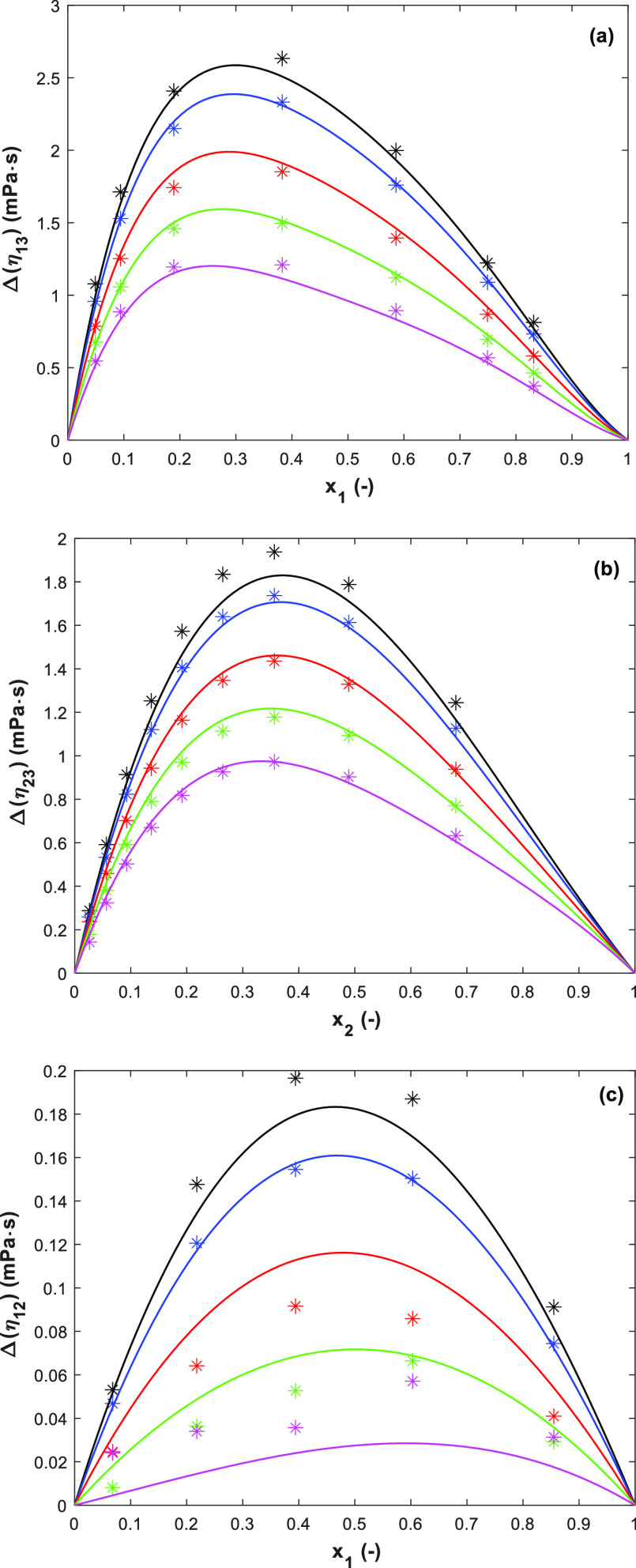
Viscosity deviations Δ(η) for (a) binary 1-(2HE)PRLD(1)/H_2_O(3), (b) binary 3A1P(2)/H_2_O(3), and (c) binary 1-(2HE)PRLD(1)/3A1P(2) at *T* = 293.15 to 363.15 K at ambient pressure (points, calculated from the measured data; lines, the RK prediction (eq 5); —, 293.15 K; blue —, 303.15 K; red —, 323.15 K; green —, 343.15 K; pink —, 363.15 K).

The representations of the viscosity deviations Δη and the RK correlation are shown, respectively, in Figure 5a for 1-(2HE)PRLD(1)/H_2_O(3), 7(b) for 3A1P(2)/H_2_O(3), and 7(c) for 1-(2HE)PRLD(1)/3A1P(2). The fitted parameters for the RK correlation are summarized in Table 8. The comparison of the model to literature data^14^ for 3A1P(2)/H_2_O(3) is also available in the Supporting Information (see Figure S10).

**Table 8:** Fitted RK Parameters for the Viscosity Deviations

coefficient		1-(2HE)PRLD(1)/H_2_O(3)		3A1P(2)/H_2_O(3)		1-(2HE)PRLD(1)/3A1P(2)		1-(2HE)PRLD(1)/3A1P(2)/H_2_O(3)	
*A*_*i*_/*B*_*i*_	*k*_*i*_	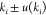	*p*_value_^*i*^	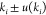	*p*_value_^*i*^	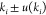	*p*_value_^*i*^	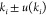	*p*_value_^*i*^
1	1	30.06 ± 0.5	0.0000	20.61 ± 0.3	0.0000	3.325 ± 0.09	0.0000	–20.5 ± 6	0.0014
	2	–0.0722 ± 0.001	0.0000	–0.0472 ± 0.001	0.0000	–0.00885 ± 0.0003	0.0000	0.042 ± 0.02	0.0294
2	3	–19.52 ± 2	0.0000	–11.26 ± 1	0.0000	–0.72 ± 0.2	0.0021		
	4	0.0459 ± 0.005	0.0000	0.0245 ± 0.003	0.0000	0.00211 ± 0.0007	0.0033		
3	5	9.9 ± 2	0.0000	–3.1 ± 2	0.0641				
	6	–0.0185 ± 0.006	0.0013	0.0124 ± 0.005	0.0148				
4	7	–11.5 ± 4	0.0069						
	8	0.0235 ± 0.01	0.0688						
RMSE		0.04		0.05		0.01		0.05	
AARD (%)		3.9		3.1		2.0		4.3	

The Δη show positive values in the entire investigated temperature and concentration ranges and decrease with increasing temperatures. The positive value usually pairs with the negative excess of the molar volume where the chemical and structural characteristics dominate. A more positive value shows that the viscosity of the real mixture is higher than that of the ideal solution in which the molecules are getting closer to each other and have less space due to strong chemical interactions and a compact structure. The decreasing Δη value with increasing temperatures indicates that the chemical interaction becomes weaker due to faster molecule movement. The maximum Δη value indicates the strongest interaction between components.

**Figure 6:**
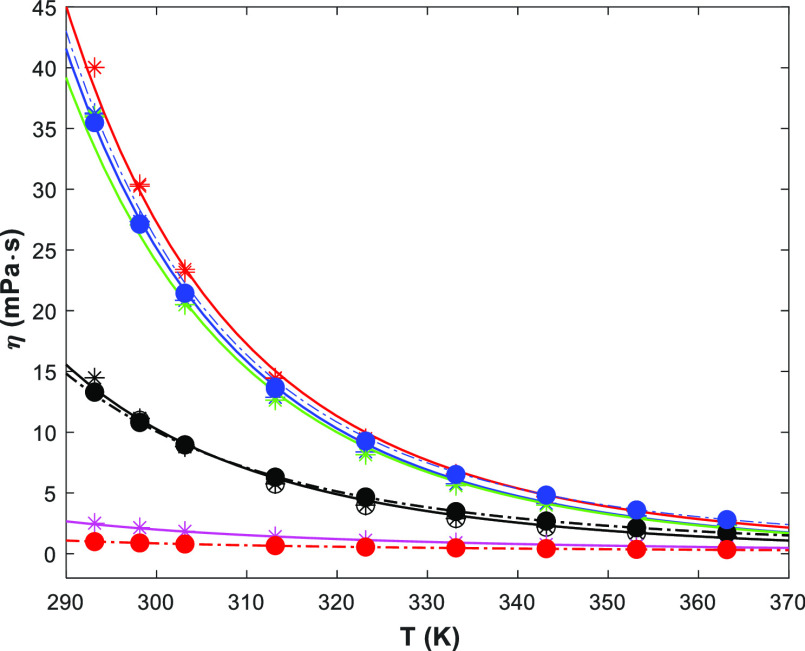
Viscosities of the ternary 1-(2HE)PRLD(1)/3A1P(2)/H_2_O(3) system from 293.15 to 363.15 K at ambient pressure (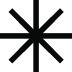/◯/—, ; blue 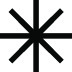/blue —, ; red 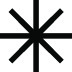/red —, ; green 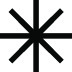/green —, ; pink 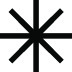/pink —, ; ●/•-•, *x*_1_ = 1.0; blue ●/blue •-•, *x*_2_ = 1.0; red ●/red •-•, *x*_3_ = 1.0; lines, eq 6).

From the fitted parameters of the RK model, the viscosities of the considered system are shown in Figures S11–S13 in the Supporting Information and for ternary 1-(2HE)PRLD(1)/3A1P(2)/H_2_O(3) in Figures 6 and S14 in the Supporting Information The agreement between the model and the viscosity data is good, and three observations can be made:

(i) when the total amine concentration is low (pink 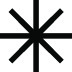), the viscosity of the solution is slightly higher than that of H_2_O.
(ii) When the total amine concentration (blue 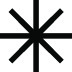/red 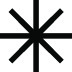/green 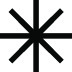) is high, the viscosity is similar to that of pure 3A1P.
(iii) Between these points with medium total amine concentration (black 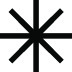), the viscosity is similar to that of pure 1-(2HE)PRLD.

## 4 Conclusions

A DMA4500 density meter coupled with a Lovis ME2000 viscosity meter was used to measure the densities and viscosities of pure 1-(2HE)PRLD, 3A1P, H_2_O, and their blends from 293.15 to 363.15 K at ambient pressure. The excess molar volumes show a negative volume due to a volume contraction for aqueous binary solutions. Intermolecular interactions and packing effects for different molecular sizes can explain this behavior. The negative values of the excess molar volume pair with the positive value of the viscosity deviations, indicating a positive deviation from its ideal mixture.

The excess molar volume and the viscosity deviation are correlated to the RK model, and the fitted parameters are reported. The agreement between the data and correlations is good, and the correlations can be used to correlated viscosity and density as a function of temperature and concentration.

## Supporting Information Available

Measured densities and viscosities of 30 mass % of MEA at different temperatures; correlation parameters for pure density and viscosity; arrangement of the DMA 4500M densitometer, Lovis 2000ME viscometer, and Xsample452; measured densities and viscosities of H_2_O; measured densities and viscosities of 30 mass % of MEA densities and H_2_O viscosities; deviation values from the measured density and viscosity compared to literature data of 30 mass % (MEA); excess volumes and measured densities for the binary 3A1P(2)/H_2_O(3) system from the literature; parity plot and the measured densities data from 293.15 to 363.15 K at ambient pressure for binary 1-(2HE)PRLD(1)/H_2_O(2), binary 3A1P(2)/H_2_O(3), binary1-(2HE)PRLD(1)/3A1P(2), and ternary 1-(2HE)PRLD(1)/3A1P(2)/H_2_O(3); viscosity deviation and the measured viscosities of binary 3A1P(2)/H_2_O(3) from the literature; and parity plots and measured viscosities from 293.15 to 363.15 K at ambient pressure of binary 1-(2HE)PRLD(1)/H_2_O(3), binary 3A1P(2)/H_2_O(3), binary 1-(2HE)PRLD(1)/3A1P(2), and ternary 1-(2HE)PRLD(1)/3A1P(2)/H_2_O(3) (PDF)
