# Supplementary material for: Densities, Viscosities of Pure 1-(2-Hydroxyethyl) Pyrrolidine, 3-Amino-1-Propanol, Water, and Their Mixtures at 293.15 to 363.15 K and Atmospheric Pressure
Source: J Chem Eng Data. 2023 Feb 23;68(3):525–35. doi: 10.1021/acs.jced.2c00648 (PMC10009753; doi:10.1021/acs.jced.2c00648)
Supplement: Supplementary file 1 — je2c00648_si_001.pdf [file je2c00648_si_001.pdf]

Densities and viscosities of pure 1-(2-Hydroxyethyl)pyrrolidine, 3-Amino-1-propanol, water and their mixtures at 293.15 to 363.15K and atmospheric pressure

Ardi Hartono and Hanna K. Knuutila

Department of Chemical Engineering, Norwegian University of Science and Technology,

N-7491 Trondheim, Norway

Supporting information

This material is available free of charge. Supporting information available:

Table S1. Measured densities and viscosities of 30 mass% of MEA ($x_{MEA} = 0.1124$) at different temperatures and at atmospheric pressure (101 kPa)[a]

T/K	$\rho/kg \cdot m^{-3}$	$\eta/mPa \cdot s$
293.15	1012.68	2.88
298.15	1010.55	2.45
303.15	1008.31	2.13
313.15	1003.45	1.63
323.15	998.07	1.31
333.15	992.23	1.06
343.15	985.96	0.88
353.15	979.27	0.74

[a]Standard uncertainties u are $u(P) = 0.3 kPa$, $u(T) = 0.01 K$, $u(x_i) = 0.0002$, $u(\eta) = 0.3\ mPa \cdot s$ for $\eta \leq 10\ mPa \cdot s$ and $u(\eta) = 0.6\ mPa \cdot s$ for $\eta > 10\ mPa \cdot s$ and $U_C(\rho) = 0.8\ kg \cdot m^{-3}$ with 0.95 level of confidence (k≈2)

Table S2. Correlation parameters for pure density (equation 3) and viscosity (equation 4):

Substance	a_i	b_i	c_i	d_i	$AARD_i$ (%)
	Density				
1-(2-Hydroxyethyl)pyrrolidine	995.28	-0.8281	0	0	0.02
3-Amino-1-propanol	1003.44	-0.7944	0	0	0.02

	Viscosity				
1-(2-Hydroxyethyl)pyrrolidine	-6.0213	4367.0086	-2.0583	0.01838	1.0
3-Amino-1-propanol	-8.4455	6250.0004	-3.2957	0.03221	2.8

Figures

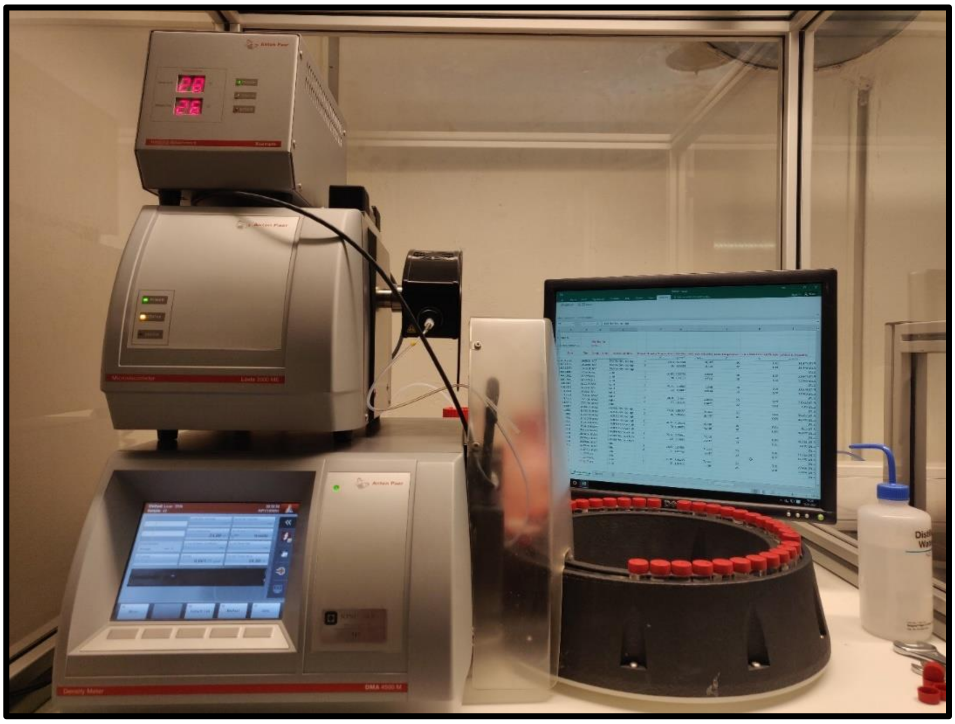

Figure S1. An arrangement of the DMA 4500M densitometer, Lovis 2000ME viscometer and Xsample452

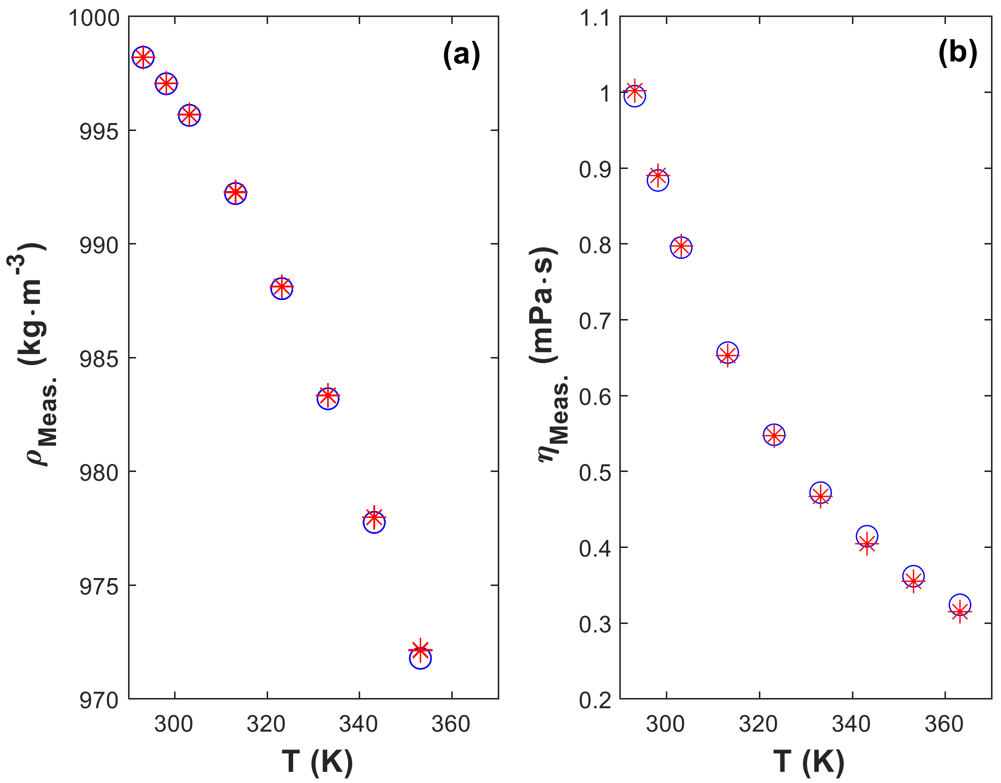

Figure S2. Measured water densities (a) (○, This work; ✳, (Spieweck and Bettin 1992)) and water viscosities (b) (○, This work; ✳, (Kestin, et al. 1978))

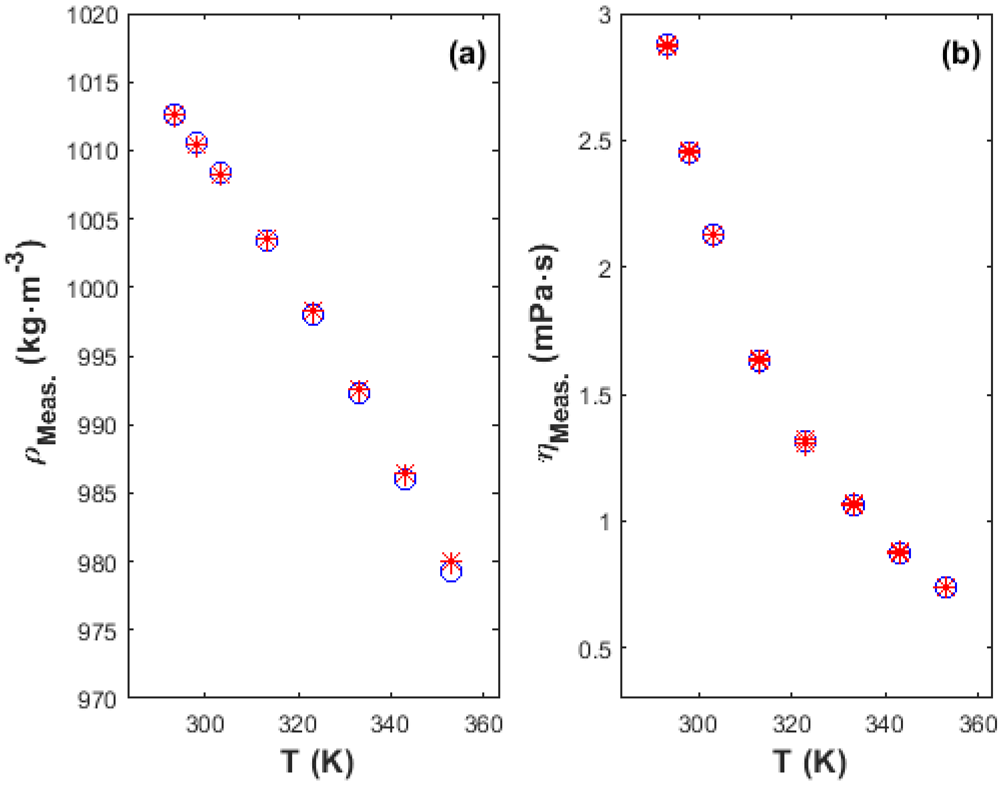

Figure S3. Measured densities (a) and viscosities (b) of 30 mass% of MEA (○, This work; ✳, (Hartono, et al. 2014)).

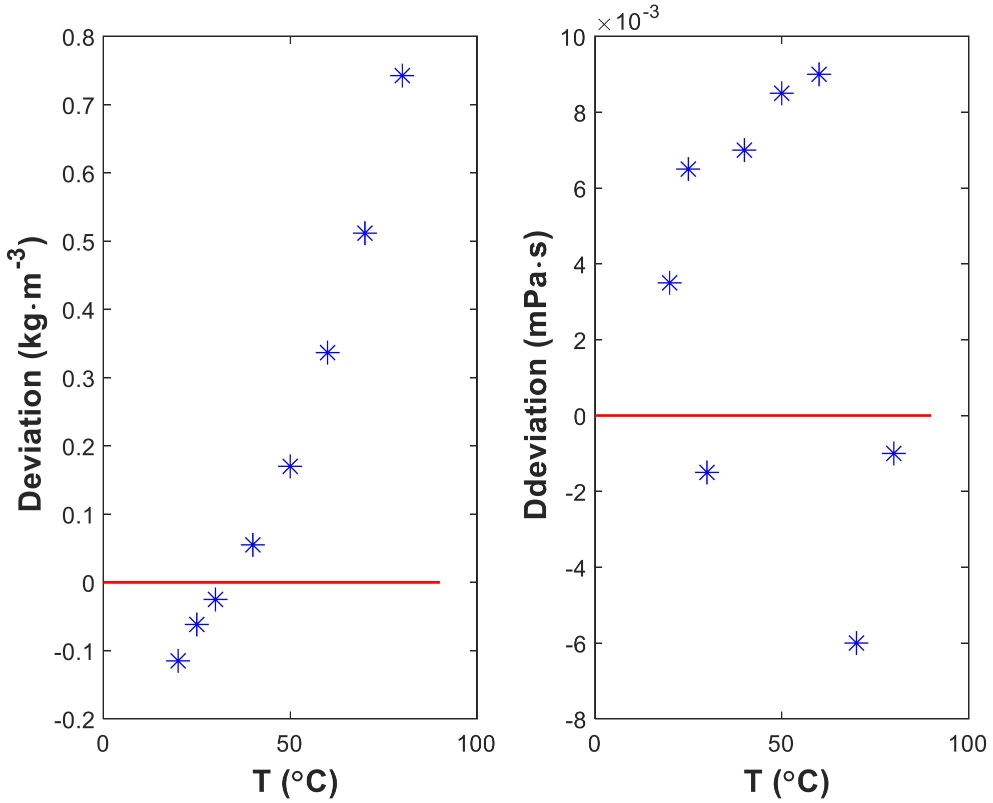

Figure S4 Deviation between the measured density and viscosity compared to literature data (Hartono, et al. 2014) of 30 mass % Monoethanolamine (MEA).

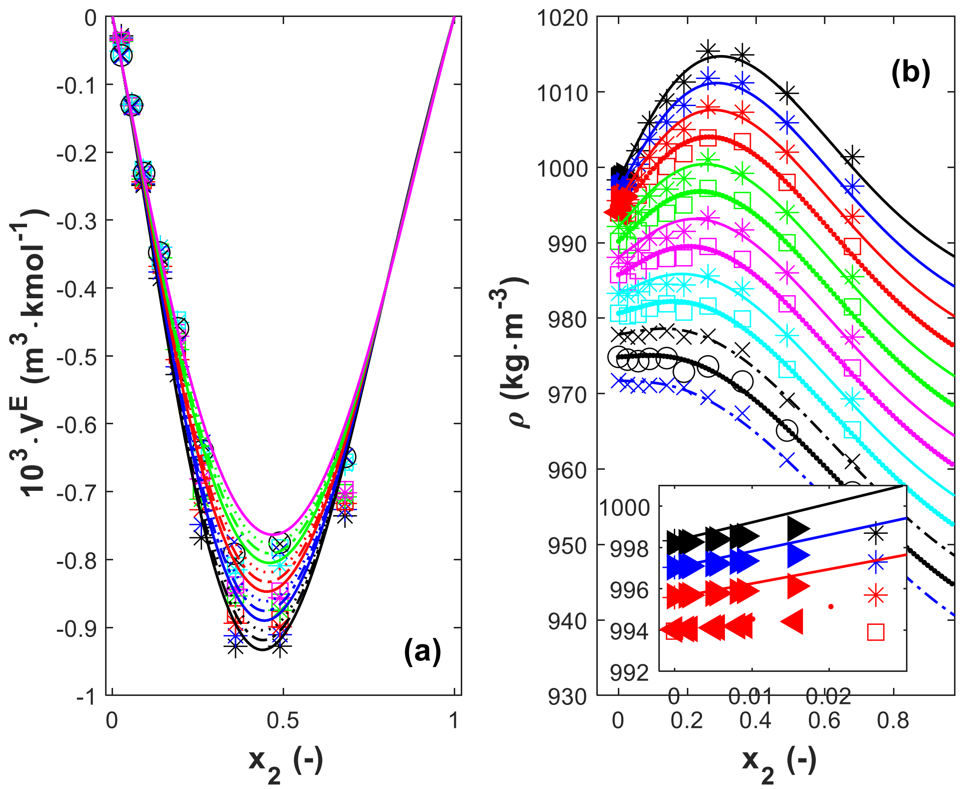

Figure S5 Excess volumes (a) and measured densities (b) for the binary 3-Amino-1-propanol(2)/ Water(3) system from 293.15 to 363.15K at ambient pressure (Points; Calculated from the measured data (a) and Experimental data (b) ; Lines, Model prediction (Equation 5);

(✳/▶/—, 293.15K; ✳/▶/—, 298.15K; ✳/▶/—, 303.15K; □/◀/—, 308.15K; ✳/—, 313.15K; □/—, 318.15K; ✳ /—, 323.15K; □/—, 328.15K; ✳ /—, 333.15K; □/—, 338.15K; ×/—•, 343.15K; O/—, 348.15K; ×/—•, 353.15K; Solid/ dotted lines, Equation 5; (✳/□/×/O), (Idris and Eimer 2016); ▶/◀, (Cruz, et al. 2021))

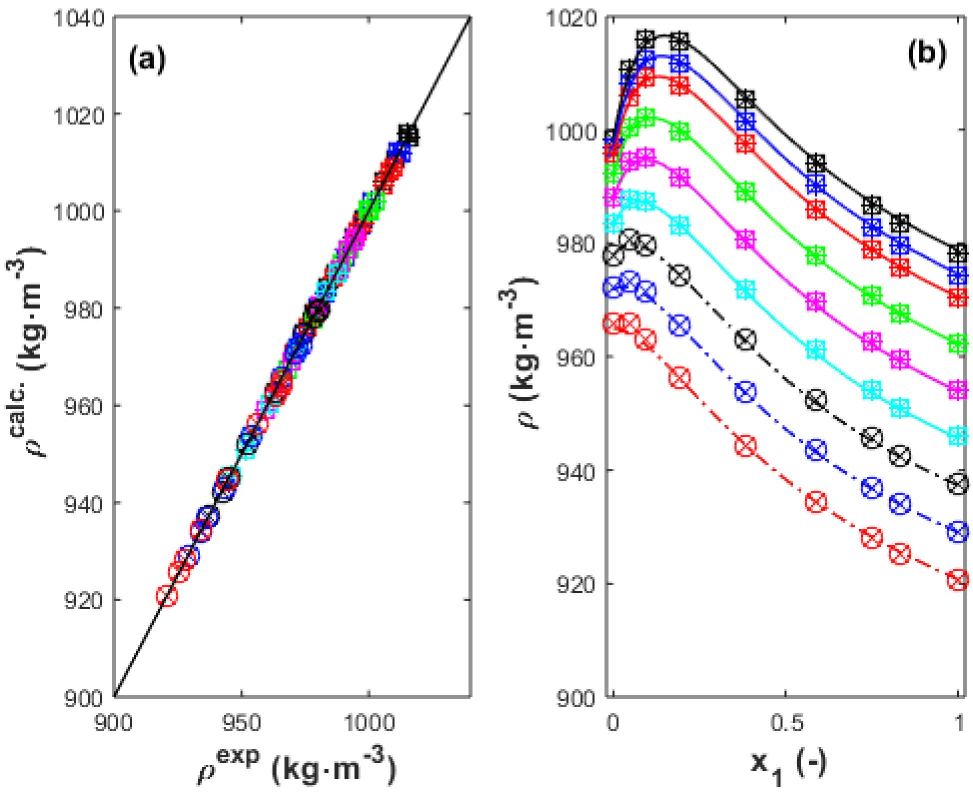

Figure S6 Parity plot (a) and the measured densities data (b) for the binary 1-(2-Hydroxyethyl)pyrrolidine(1)/ water(2) from 293.15 to 363.15K at ambient pressure (✳/□, 293.15K; ✳/□, 298.15K; ✳/□, 303.15K; ✳/□, 313.15K; ✳/□, 323.15K; ✳/□, 333.15K; ×/O 343.15K; ×/O, 353.15K; ×/O, 363.15K; Solid/ dotted lines, Equation 5; Points, This work)

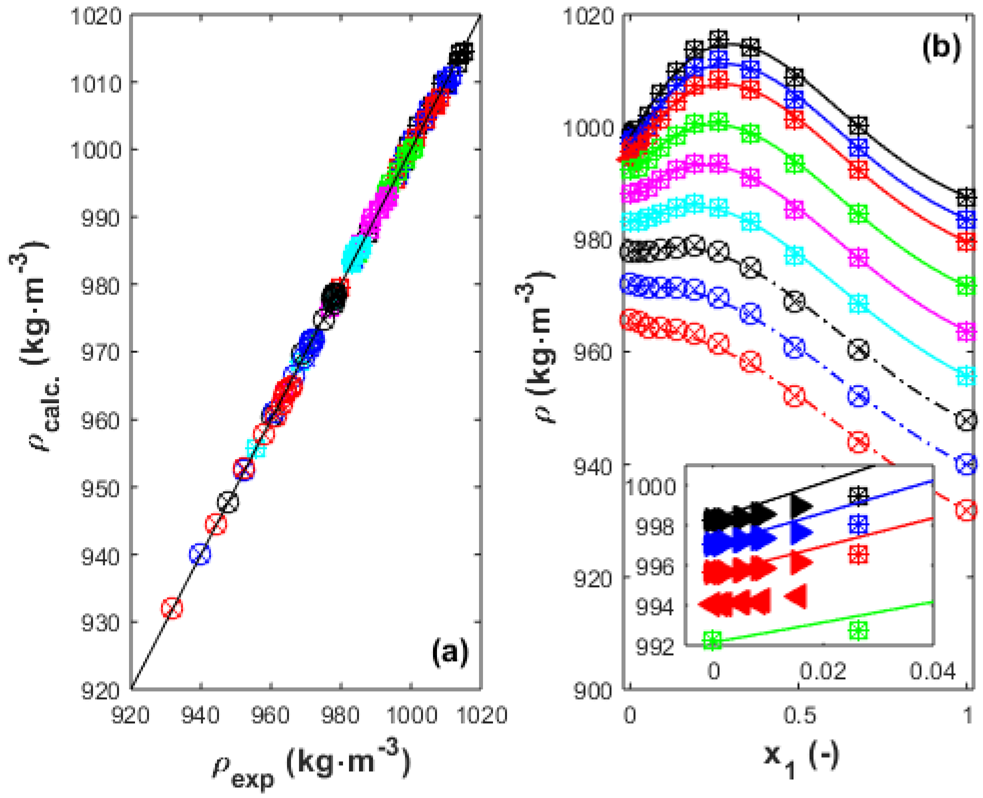

Figure S7 Parity plot (a) and measured densities (b) for the binary 3-Amino-1-propanol(2)/ water(3) from 293.15 to 363.15K at ambient pressure (✳/□/▶, 293.15K; ✳/□/▶, 298.15K; ✳/□/▶, 303.15K; ◀, 308.15K;✳/□, 313.15K; ✳/□, 323.15K; ✳/□, 333.15K; ×/O, 343.15K; ×/O, 353.15K; ×/O, 363.15K; ▶/◀, (Cruz, et al. 2021); ✳/□/×/O, This work; Solid/ dotted lines, Equation 5)

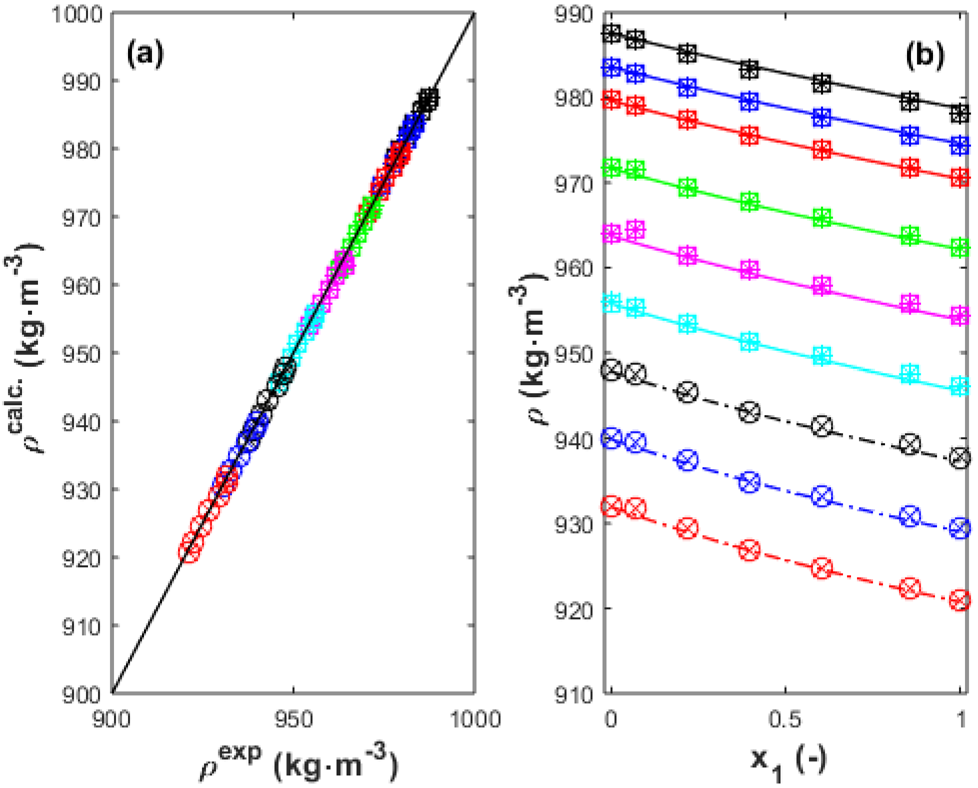

Figure S8 Parity plot (a) and ratio of the calculated to the measured (b) densities of the binary of 1-(2-Hydroxyethyl)pyrrolidine(1)/ 3-Amino-1-propanol(2) from 293.15 to 363.15K at ambient pressure (✳/□, 293.15K; ✳/□, 298.15K; ✳/□, 303.15K; ✳/□, 313.15K; ✳/□, 323.15K; ✳/□, 333.15K; ×/O, 343.15K; ×/O, 353.15K; ×/O, 363.15K; ✳/□/×/O, This work; Solid/ dotted lines, Equation 5)

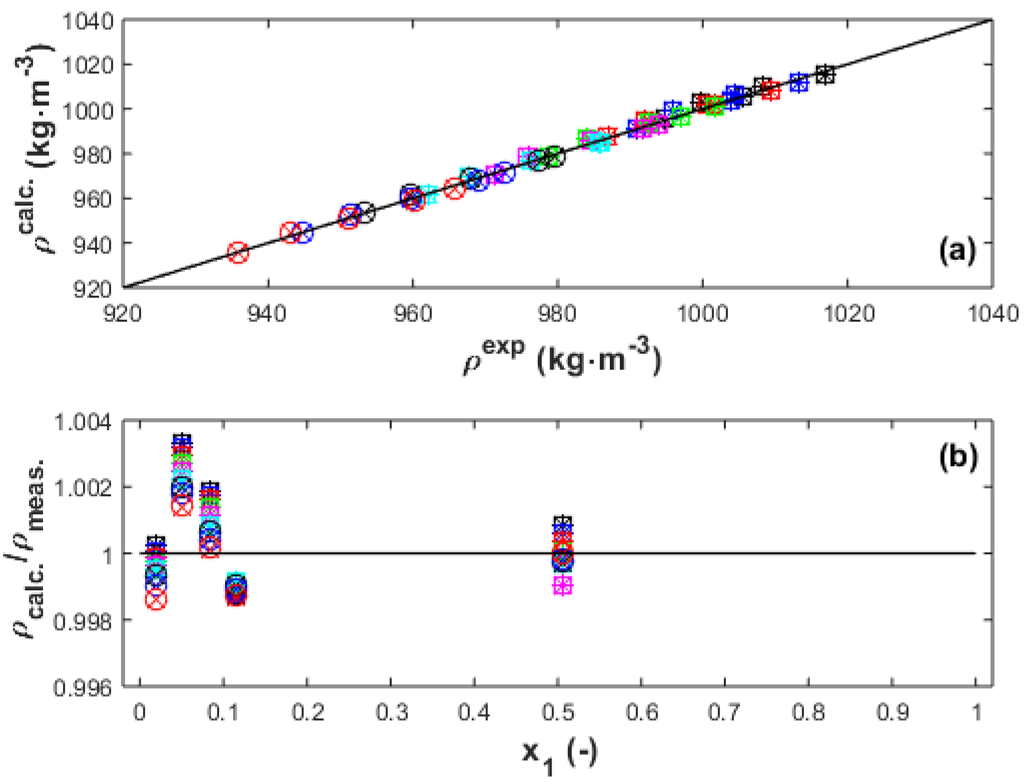

Figure S9 Parity plot (a) and ratio of the calculated to the measured (b) densities of the ternary of 1-(2-Hydroxyethyl)pyrrolidine(1)/ 3-Amino-1-propanol(2)/ water(3) system from 293.15 to 363.15K at ambient pressure (✳/○, $\frac{x_1}{x_2} = \frac{0.1148}{0.0667}$; ✳/○, $\frac{x_1}{x_2} = \frac{0.0831}{0.3820}$; ✳/○, $\frac{x_1}{x_2} = \frac{0.0504}{0.6185}$; ✳/○, $\frac{x_1}{x_2} = \frac{0.5063}{0.0991}$; ✳/○, $\frac{x_1}{x_2} = \frac{0.0189}{0.0320}$; ●, $\frac{x_1}{x_2} = \frac{1.0000}{0.0000}$; ●, $\frac{x_1}{x_2} = \frac{0.0000}{1.0000}$; ●, $\frac{x_1}{x_2} = \frac{0.0000}{0.0000}$)

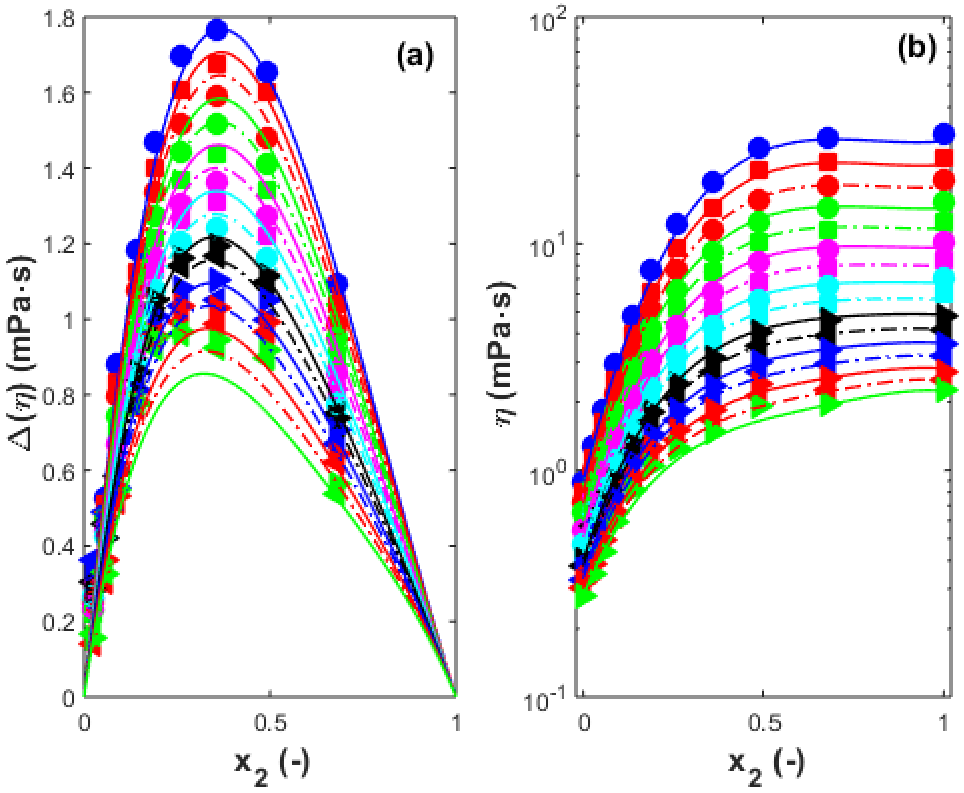

Figure S10 Viscosity deviation (a) and the measured (b) viscosities of the binary 3-Amino-1-propanol(2)/ water(3) from 298.15 to 373.15K at ambient pressure (●, 298.15K; ■, 303.15K; ●, 308.15K; ■, 313.15K; ●, 318.15K; ■, 323.15K; ●, 328.15K; ■, 333.15K; ●, 338.15K; ▶, 343.15K; ◀, 348.15K; ▶, 353.15K; ◀, 358.15K; ▶, 363.15K; ◀, 368.15K; ▶, 373.15K; lines on η (mPa.s), Equation 5; Data, (Idris, et al. 2018))

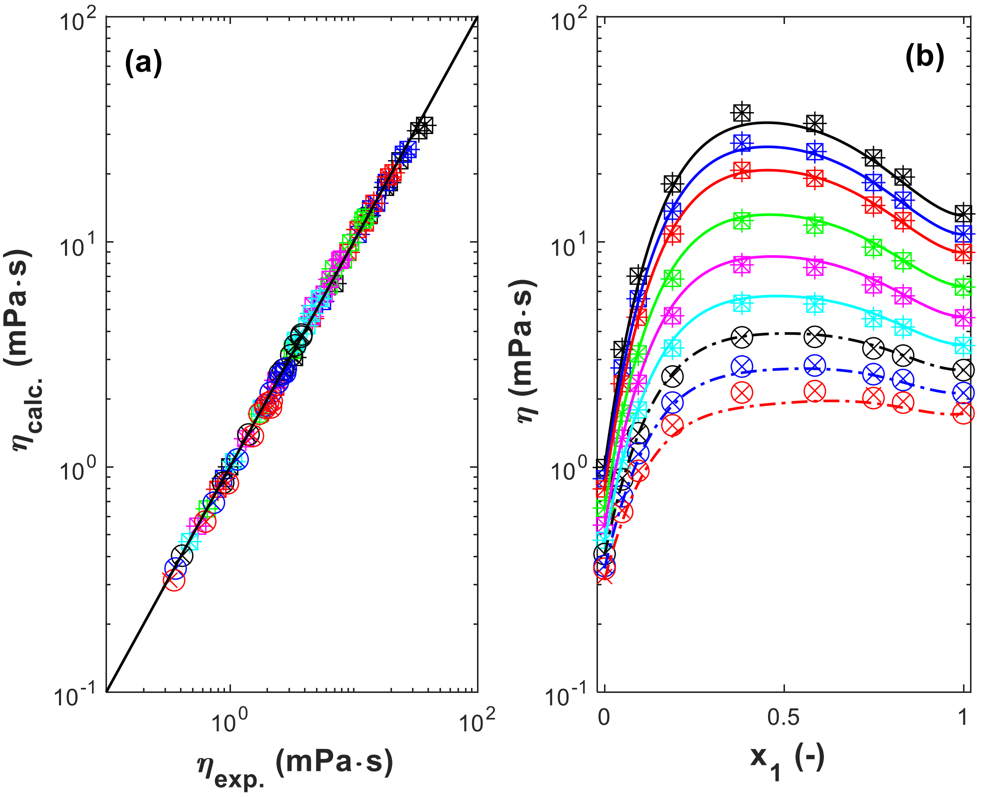

Figure S11 Parity plot (a) and the measured viscosities (b) for the binary 1-(2-Hydroxyethyl)pyrrolidine(1)/ water(3) from 293.15 to 363.15K at ambient pressure (✳/□, 293.15K; ✳/□, 298.15K; ✳/□, 303.15K; ✳/□, 313.15K; ✳/□, 323.15K; ✳/□, 333.15K; ×/○•, 343.15K; ×/○, 353.15K; ×/○, 363.15K; ✳/□/×/○, This work; Solid/ dotted lines, Equation 5)

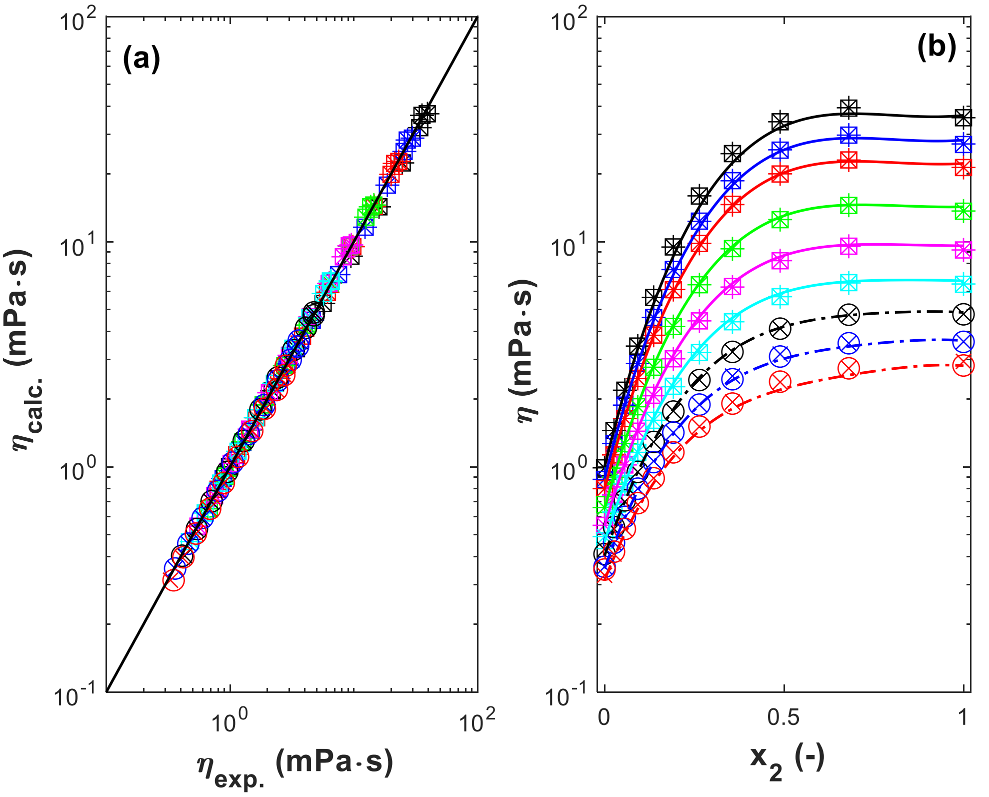

Figure S12 Parity plot (a) and the measured viscosities (b) for the binary 3-Amino-1-propanol(2)/ water(3) from 293.15 to 363.15K at ambient pressure (✳/□, 293.15K; ✳/□, 298.15K; ✳/□, 303.15K; ✳/□, 313.15K; ✳/□, 323.15K; ✳/□, 333.15K; ×/O, 343.15K; ×/O, 353.15K; ×/O, 363.15K; ✳/□/×/O, This work; Solid/ dotted lines, Equation 5)

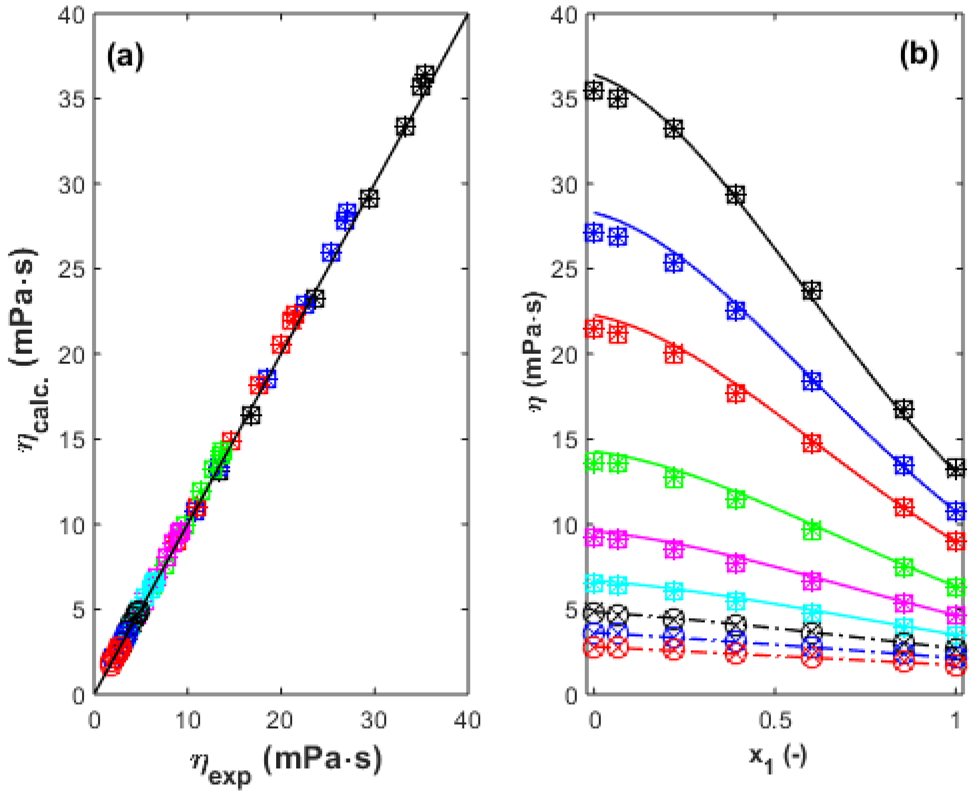

Figure S13 Parity plot (a) and ratio of the calculated to the measured (b) viscosities of binary of 1-(2-Hydroxyethyl) pyrrolidine(1)/ 3-Amino-1-propanol(2) from 293.15 to 363.15K at ambient pressure (✳/□, 293.25K; ✳/□, 298.15K; ✳/□, 303.15K; ✳/□, 313.15K; ✳/□, 323.15K; ✳/□, 333.15K; ×/O, 343.15K; ×/O, 353.15K; ×/O, 363.15K; ✳/□/×/O, This work; Solid/ dotted lines, Equation 5)

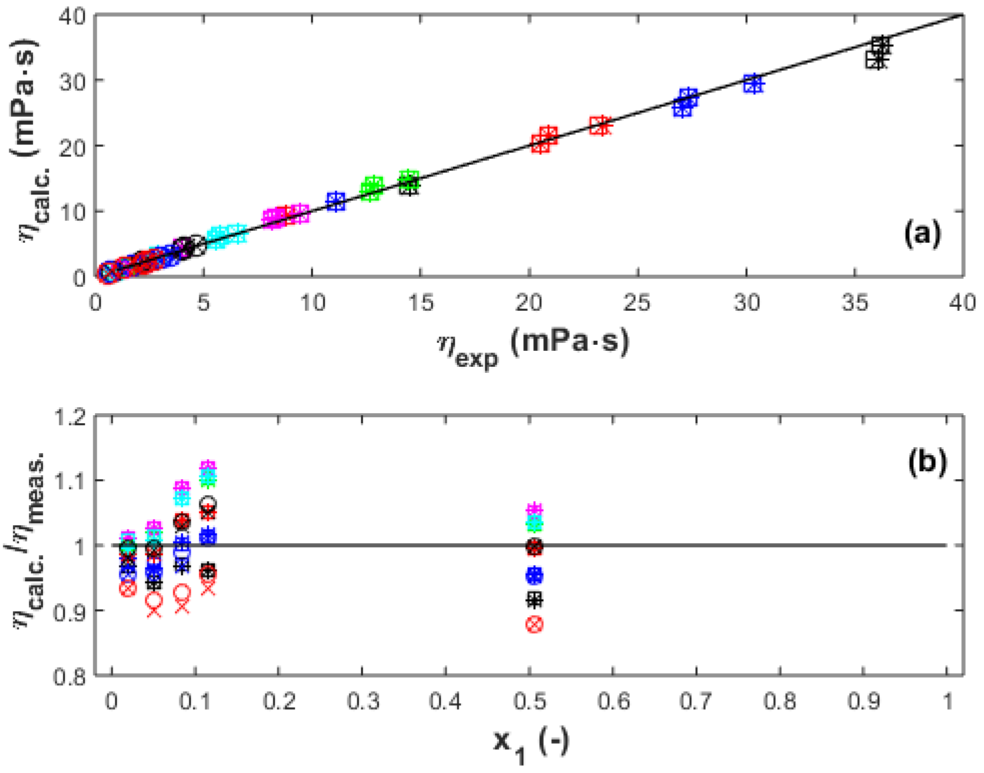

Figure S14 Parity plot (a) and ratio of the calculated to the measured (b) viscosities of the ternary of 1-(2-Hydroxyethyl)pyrrolidine(1)/ 3-Amino-1-propanol(2)/ water(3) system from 293.15 to 363.15K at ambient pressure (✳/○, $\frac{x_1}{x_2} = \frac{0.1148}{0.0667}$; ✳/○, $\frac{x_1}{x_2} = \frac{0.0831}{0.3820}$; ✳/○, $\frac{x_1}{x_2} = \frac{0.0504}{0.6185}$; ✳/○, $\frac{x_1}{x_2} = \frac{0.5063}{0.0991}$; ✳/○, $\frac{x_1}{x_2} = \frac{0.0189}{0.0320}$; ●, $\frac{x_1}{x_2} = \frac{1.0000}{0.0000}$; ●, $\frac{x_1}{x_2} = \frac{0.0000}{1.0000}$; ●, $\frac{x_1}{x_2} = \frac{0.0000}{0.0000}$)

Reference

Cruz, Yadhi P., Miguel A. Esteso, and Carmen M. Romero

2021 Effect of temperature on the partial molar volumes and the partial molar compressibilities of amino alcohols in aqueous solution. The Journal of Chemical Thermodynamics 160:106521.

Hartono, A., E. O. Mba, and H. F. Svendsen

2014 Physical properties of partially CO2 loaded aqueous monoethanolamine (MEA). Journal of Chemical and Engineering Data 59(6):1808-1816.

Idris, Zulkifli, and Dag A. Eimer

2016 Density Measurements of Unloaded and CO2-Loaded 3-Amino-1-propanol Solutions at Temperatures (293.15 to 353.15) K. Journal of Chemical & Engineering Data 61(1):173-181.

Idris, Zulkifli, Nithin B. Kummamuru, and Dag A. Eimer

2018 Viscosity Measurement and Correlation of Unloaded and CO2-Loaded 3-Amino-1-propanol Solution. Journal of Chemical & Engineering Data 63(5):1454-1459.

Kestin, Joseph, Mordechai Sokolov, and William A. Wakeham

1978 Viscosity of liquid water in the range −8 °C to 150 °C. Journal of Physical and Chemical Reference Data 7(3):941-948.

Spieweck, F., and H. Bettin

1992 Review: Solid and liquid density determination / Übersicht: Bestimmung der Dichte von Festkörpern und Flüssigkeiten. tm - Technisches Messen 59(7-8):285-292.
